# Control of pattern formation during phase separation initiated by a propagated trigger

**DOI:** 10.1038/s41598-017-07352-z

**Published:** 2017-07-31

**Authors:** Rei Kurita

**Affiliations:** Department of Physics, Tokyo Metropolitan University, 1-1 Minamioosawa, Hachiouji-shi, Tokyo 192-0397 Japan

## Abstract

Understanding pattern formation during phase separation is a key topic in materials science for the important role that patterns play in determining macroscopic physical properties. In this work, we show how pattern formation can be controlled using a phase-separation trigger propagating outwards from a point. We found a range of patterns, including a random droplet pattern, a concentric pattern and a dendritic pattern, depending on the speed at which the trigger propagates, while only the random droplet pattern is observed in a system with homogeneous cooling. We also found that the phase at the core of the concentric pattern periodically changes with time. In addition, we investigated pattern formation during phase separation induced by multiple propagated triggers. When we propagate the triggers from periodic points in space, a metastable regular hexagonal pattern is formed. We also found a bifurcation between a case where the majority phase becomes a droplet phase and a case where the minority phase adopts a droplet pattern. We also confirm the existence of a percolated, bicontinuous phase, even with an asymmetric composition.

## Introduction

Phase separation in two component mixtures can lead to the formation of patterns; their features are directly related to the macroscopic physical properties of the material. This connection between formation and function has motivated a significant body of research over many decades due to its importance for material science^1–11^. When a two-component mixture is quenched below the phase separation line, a bicontinuous network pattern is formed if the proportions of the phases are the same. Meanwhile, when there is a high degree of asymmetry between the volume fractions of the phases, the minority phase can appear as droplets, with random droplet size and spatial distribution. It is known that these patterns coarsen in a self-similar manner, and that the phenomenon is ubiquitous for phase separating two component mixtures. Meanwhile, pattern formation can be significantly different in polymer solutions^6, 12^. During phase separation in polymer solutions, the polymer transiently forms a network; the network then transforms into droplets in a coarsening process. This phase separation is called *viscoelastic phase separation* and occurs when the dynamics of one component is significantly slower than the other. This shows that pattern formation can depend on dynamical properties.

Such pattern formation can also be controlled by using the properties of the surface bounding a particular mixture^13–18^. If one of the two components prefers a surface, the concentration of the component becomes higher near the surface. This enhancement of concentration by wetting affects concentration fluctuations over the whole system, changing the pattern formation seen due to spinodal decomposition^13–16^. Recently, pattern formation control using Janus colloids was proposed. Janus colloids are particles whose two hemispheres have distinct physical properties; coupling between the mobility of the Janus colloids and the different wetting properties on the surfaces can induce regular lamella pattern formation^17, 18^.

Another way to control pattern formation is to change the dynamical path such as a multi step quenching^19–22^ and a directional quenching^23–26^. Furukawa proposed a way to form regular patterns using a directional quenching system^23^. Different parts of the system are quenched below a phase separation temperature, but only when a ‘trigger’ has arrived, propagating through space over time; in the case of directional quenching, the trigger, which is analogous to the temperature quenching, propagates as a linear front in one direction with constant velocity *v*_*tri*_. It is interesting to see that the pattern formed depends on *v*_*tri*_^23–26^. We can observe a random droplet pattern similar to the usual pattern seen in homogeneous quenching when *v*_*tri*_ is quite fast. A regular lamella structure (RLS) parallel to the trigger front is formed if *v*_*tri*_ is similar to the speed of phase separation. For slower *v*_*tri*_, a regular column structure (RCS) perpendicular to the quenching face is formed^23^. Recently, a re-entrance from RCS to RLS was reported if *v*_*tri*_ is significantly slower, due to macroscopic coarsening^25^. Such behaviour can be observed in polymer experiments^27, 28^. It is important to note that the patterns are different from those seen in spatially homogeneous quenching, even though the temperature becomes homogeneous in the final state.

For this work, we performed numerical simulations to investigate new pattern formation mechanisms during phase separation. Specifically, we consider a phase separation trigger propagating radially from a point, such that it induces a temperature quench on arrival at different parts of the system. Note that the space in front of and behind the trigger are asymmetric, not symmetric like for the planar trigger. Due to such symmetry breaking, we observe different pattern formation from the layered structure reported in previous studies^23–26^. In addition, we studied pattern formation with multiple triggers propagating from periodically placed points.

## Model and Methods

We used a 2-dimensional model to simulate phase separation with radial trigger propagation. We used a modified Cahn-Hiliard-Cook equation as given below^1, 29^,

1 $$\frac{\partial \varphi }{\partial t}=L{\nabla }^{2}[a|T-{T}_{c}|\sigma (\vec{r})\varphi +b{\varphi }^{3}-c{\nabla }^{2}\varphi ]+\theta $$

where *ϕ*, *L* and *θ* are concentration, a transportation coefficient and thermal noise, respectively. *a*, *b*, and *c* are positive constants. *T* is temperature and *T*_*c*_ is the phase separation temperature. $\sigma (\vec{r})$ is ±1, a control parameter for trigger propagation. The Cahn-Hiliard-Cook equation describes the dynamics of phase separation in systems with a conserved order parameter and no hydrodynamics; it is well known that this equation describes the dynamics of critical phenomenon and phase separation^4^. We note that numerical studies for directional quenching used the same equation, and that their results were consistent with experiment. Thus, we consider a numerical investigation using Eq. (1) to be appropriate.

Here, the correlation length and the characteristic time can be written as $\xi =\sqrt{c/a|T-{T}_{c}|}$ and ${\tau }_{0}=$ ${\xi }^{2}/La|T-{T}_{c}|$, respectively. We normalized the length, time and *ϕ* by *ξ*, *τ*_0_ and ${\varphi }_{0}=\sqrt{a|T-{T}_{c}|/b}$, where *ϕ*_0_ corresponds to the concentration after phase separation. We then obtain a normalized equation (1),

2 $$\frac{\partial \varphi }{\partial t}={\nabla }^{2}[\sigma (\vec{r})\varphi +{\varphi }^{3}-{\nabla }^{2}\varphi ]+{\rm{\Theta }}.$$

We set Θ to satisfy fluctuation-dissipation theory. Here, we have two controllable parameters, the sign of $\sigma (\vec{r})$ and the mean concentration $\overline{\varphi }$. The sign of $\sigma (\vec{r})$ changes with temperature, in line with realistic phase separation phenomena. $\sigma (\vec{r})$ = 1 when *T* is over *T*_*c*_; the stable phase is a homogeneous one. $\sigma (\vec{r})$ = −1 when *T* is below *T*_*c*_; in this case, phase separation occurs at $\vec{r}$.

We initially set the concentration to $\overline{\varphi }$ and ran a simulation where *σ* = 1 everywhere for a long time, to prepare an initial state. We then began to propagate a trigger radially outwards from a point with a constant speed *v*_*tri*_ and set this time to *t* = 0. The arrival of the trigger corresponds to a directional temperature quench. $\sigma (\vec{r})$ = −1 if |$|\vec{r}-{\vec{r}}_{c}| < {v}_{tri}t$, where $\vec{r}$_*c*_ is the position of the point from which the trigger is propagated; otherwise, $\sigma (\vec{r})$ = 1.

We performed numerical simulations using two conditions. (i) We propagated the trigger from the center of the simulation box to the end. The simulation box is a 512 × 512 grid with periodic boundary conditions. We defined *t*_*h*_ as the time when the trigger reaches the end of the simulation box i.e. ${t}_{h}=256\times \sqrt{2}/{v}_{tri}$ (ii) We propagated triggers from multiple spots, arranged in a hexagonal pattern with a spacing Δ*d*. We set the size of the simulation box to fulfil *n*Δ*d* × $\sqrt{3}/2n{\rm{\Delta }}d$, where *n* is an integer, such that the arrangement of the trigger generation spots is also periodic. We set *n* such that *n*Δ*d* is close to 512 and get ${t}_{h}=\sqrt{3}{\rm{\Delta }}d/2{v}_{tri}$. In both simulations, temperature is under *T*_*c*_ everywhere after *t* = *t*_*h*_. Our study thus corresponds to a study of pattern formation following different dynamical paths at the early stages of phase separation.

## Pattern formation with a single trigger

**Figure 1:**
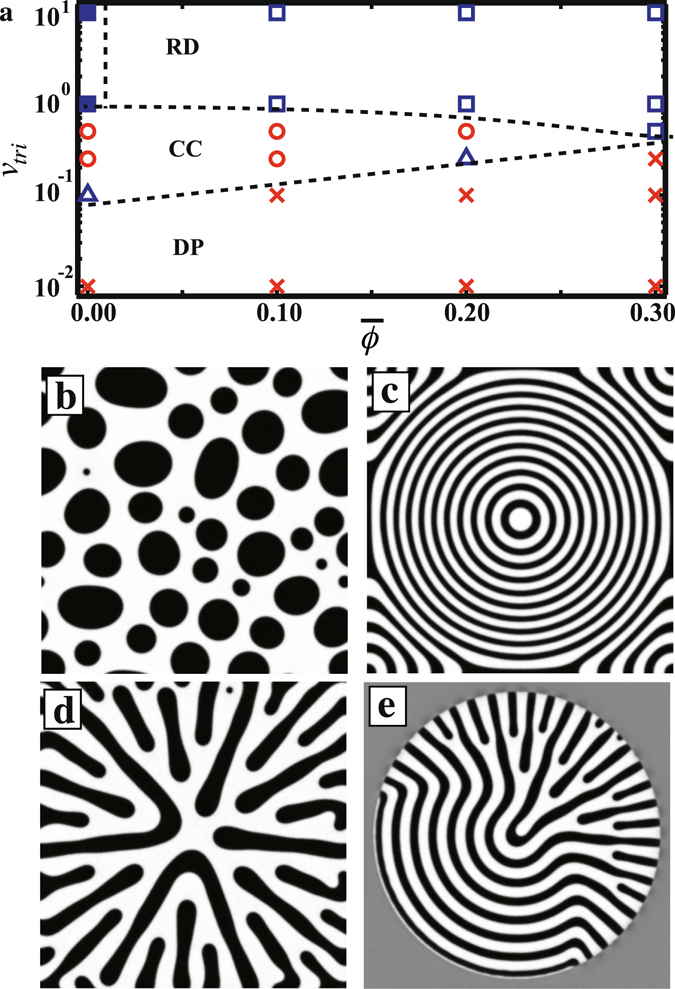
Pattern formation during phase separation. (**a**) Transient patterns formed during phase separation as functions of $\overline{\varphi }$ and *v*_*tri*_. The symbols in (**a**) are the simulated points. Filled square, open square, open circle, and cross symbols correspond to a bicontinuous pattern, random droplet pattern, concentric circles, and dendritic pattern, respectively. The triangle symbol corresponds to a mixture of concentric circles and a dendritic pattern. The dashed lines are guides separating regions where different transient patterns are observed. (**b**) A random droplet pattern is observed when $\overline{\varphi }$ = 0.1, *v*_*tri*_ = 10 and *t* = 1500. (**c**) A concentric circle pattern is observed when $\overline{\varphi }$ = 0.1, *v*_*tri*_ = 0.5 and *t* = 1500. (**d**) A dendritic pattern is observed when $\overline{\varphi }$ = 0.1, *v*_*tri*_ = 0.1 and *t* = 4000. We note that (**b**–**d**) are images after the trigger has covered the entire system. (**e**) A combination of concentric circle and dendritic patterns is observed when $\overline{\varphi }$ = 0, *v*_*tri*_ = 0.1 and *t*  = 2000.

Firstly, we show what kind of patterns are transiently formed at the beginning of the phase separation for different $\overline{\varphi }$ and *v*_*tri*_ [Fig. 1(a)]. Each point corresponds to an independent simulation. When the phases are symmetric i.e. $\overline{\varphi }$ = 0, a bicontinuous pattern (filled square) is formed when 1 ≤ *v*_*tri*_ ≤ 10. We also observe a random droplet pattern (RD), where the spatial arrangement and size of droplets is random, at $\overline{\varphi }$ = 0.1 with *v*_*tri *_= 10 as shown in Fig. 1(b). White in Fig. 1(b–e) corresponds to *ϕ* = 1, the majority phase; black corresponds to *ϕ* = −1. It is similar to the pattern seen for homogeneous quenching since *v*_*tri*_ is much larger than the characteristic speed of the phase separation *v*_0_ (=*ξ*/*τ*_0_ = 1). When *v*_*tri*_ = 0.5, slightly slower than *v*_0_, the pattern takes the form of concentric circles (CC) [Fig. 1(c)]. This pattern is parallel to the trigger front and is consistent with previous findings^24^. In addition, the CC pattern is similar to the pattern which is induced by surface-directed spinodal decomposition^13, 14^. As we discuss later, there are clear differences between the trigger propagated system and surface-directed spinodal decomposition. We also find that a dendritic pattern (DP) is formed for *v*_*tri*_ ≤ 0.1 [Fig. 1(d)]. The DP pattern corresponds to the RCS seen in the directional quenching system^23^. The triangle in Fig. 1(a) corresponds to a mixture of the CC pattern and the DP pattern [Fig. 1(e)]. The DP pattern can also be observed when particles aggregate in response to a propagating trigger in a dense particulate system^30^. The transient patterns seen at the beginning of the phase separation are also given; the dashed lines in Fig. 1(a) are visual guides for the location of boundaries. Here, we see that the DP pattern can be observed for 0 ≤ $\overline{\varphi }$ ≤ 0.3. Also note that the region where a CC pattern is seen in the diagram becomes narrower with increasing $\overline{\varphi }$. We find that the width of the circular ring containing the minority (black) phase in Fig. 1(c) becomes thinner with increasing $\overline{\varphi }$. When $\overline{\varphi }$ > 0.3, we find that the circular rings are unstable due to a Plateau-Rayleigh instability; thus, a CC pattern cannot be observed in this regime.

**Figure 2:**
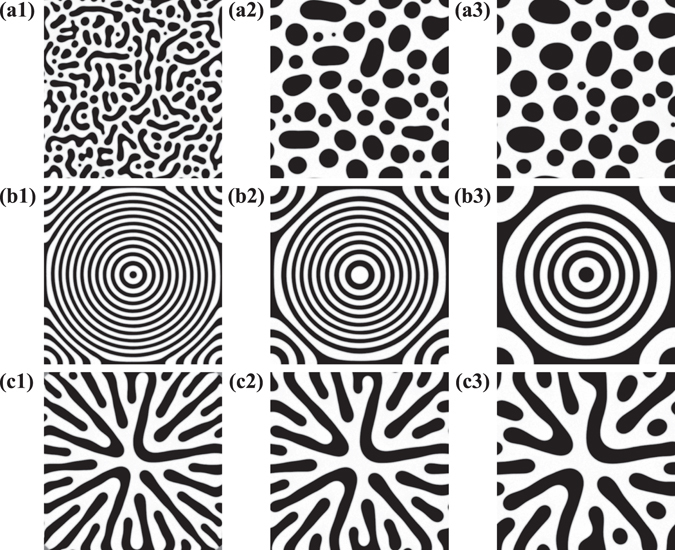
Time evolution of transient patterns with a single propagated trigger when $\overline{\varphi }$ = 0.1. (**a**) Time evolution of the random droplet pattern when *v*_*tri*_ = 10 at *t* = 100 (a1), 1000 (a2) and 10000 (a3). (**b**) Time evolution of the CC pattern when *v*_*tri*_ = 0.5 at *t* = 1000 (b1), 5000 (b2) and 20000 (b3). (**c**) Time evolution of the DP pattern when *v*_*tri*_ = 0.1 at *t* = 4000 (c1), 8000 (c2) and 15000 (c3). All images correspond to times after the trigger has reached the end of the simulation box.

**Figure 3:**
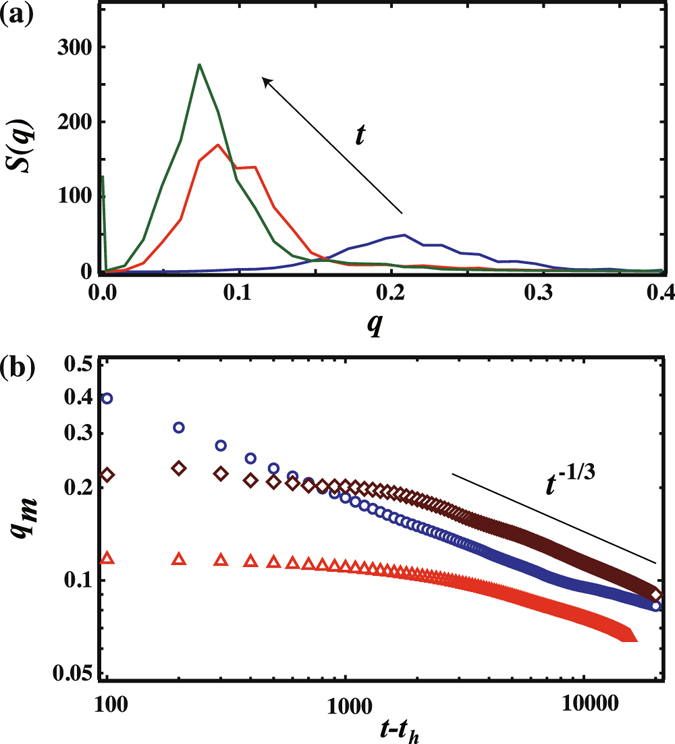
(**a**) Structure factor *S*(*q*) for the random droplet pattern when *v*_*tri*_ = 10, observed over time. We show *S*(*q*) at *t* = 800, 5000 and 20000. (**b**) Time evolution of the peak position *q*_*m*_ as a function of *t* − *t*_*h*_. *t*_*h*_ is the time after the trigger has covered the whole box, and the system is homogeneously quenched. The circle symbol corresponds to *q*_*m*_ for the random droplet pattern. Diamond and triangle symbols correspond to *q*_*m*_ for concentric circles and the dendritic pattern, respectively. The solid line in (**b**) shows (*t* − *t*_*h*_)^−1/3^.

We go on to investigate the time evolution of the patterns when $\overline{\varphi }$ = 0.1. During the coarsening process, small domains are absorbed into large domains in such a way as to reduce the interfacial energy. In RD patterns when *v*_*tri*_ = 10, the large droplets coarsen with absorption of the small droplets as shown in Fig. 2(a1–a3). These images corresponds to times after the temperature quench has covered the entire simulation box. We obtain a structure factor *S*(*q*) by taking a Fourier transform of the image shown in Fig. 3(a). We can then compute a peak position using *S*(*q*), *q*_*m*_, using the equation ${q}_{m}=\int qS(q)dq/\int S(q)dq$. We show the time evolution of *q*_*m*_ after the trigger has covered the entire box in Fig. 3(b), and find that the characteristic size grows following a *t* ^1/3^ law which is consistent with previous studies^4^.

**Figure 4:**
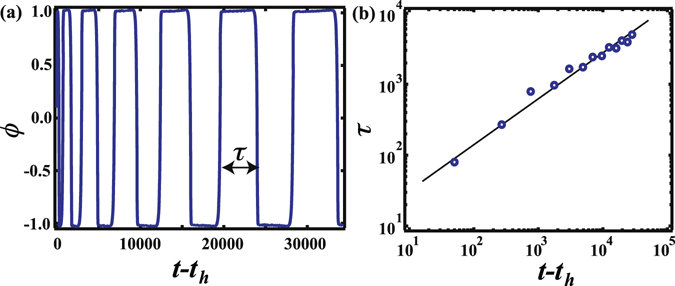
(**a**) Time evolution of *ϕ* at the core of a concentric circle pattern when $\overline{\varphi }$ = 0.1 and *v*_*tri*_ = 0.5. *ϕ* at the core is oscillating between *ϕ* = 1 and *ϕ* = −1. *τ* corresponds to a cycle of *ϕ* evolution at the core. (**b**) *τ* as a function of *t*. *τ* increases with time following a *t* ^0.62^ power law (solid line).

Meanwhile, the coarsening dynamics of the CC pattern is shown in Fig. 2(b1–b3). The phase at the core of the CC pattern repeatedly switches between white and black. The time evolution of *ϕ* at the core of the CC pattern is given in Fig. 4(a). We find that the *ϕ* value at the core is oscillating between *ϕ* = 1 and *ϕ* = −1. Here, we note that the area of the core is smaller than that of the neighbouring circular ring. Thus, the domain at the core becomes smaller over time while the neighbouring circular ring becomes wider. The ring next to the core goes on to become the new core after the previous core vanishes. At the same time, the number of circular rings decreases by one. This behaviour occurs repeatedly until the circular rings become one droplet. Figure 4(b) shows a single cycle *τ* over which *ϕ* changes at the core as a function of *t* − *t*_*h*_. We find that *τ* increases following a power law, (*t* − *t*_*h*_)^0.62^. The origin of this exponent is yet to be determined. We also show the time evolution of *q*_*m*_ after *t* = *t*_*h*_, when the trigger reaches the end of the simulation box [Fig. 3(b)]. *q*_*m*_ is almost constant below *t* − *t*_*h*_ = 2000 and then decreases following (*t* − *t*_*h*_)^−1/3^.

Moving on to the dendritic pattern, we find that the DP pattern coarsens through self-assembly over time as shown in Fig. 2(c1–cs3). We again show the time evolution of *q*_*m*_ as a function of *t* − *t*_*h*_ [Fig. 3(b)]. *q*_*m*_ is almost constant below *t* − *t*_*h*_ = 1500 and then decreases following (*t* − *t*_*h*_)^−1/3^.

## Pattern formation with multiple triggers

**Figure 5:**
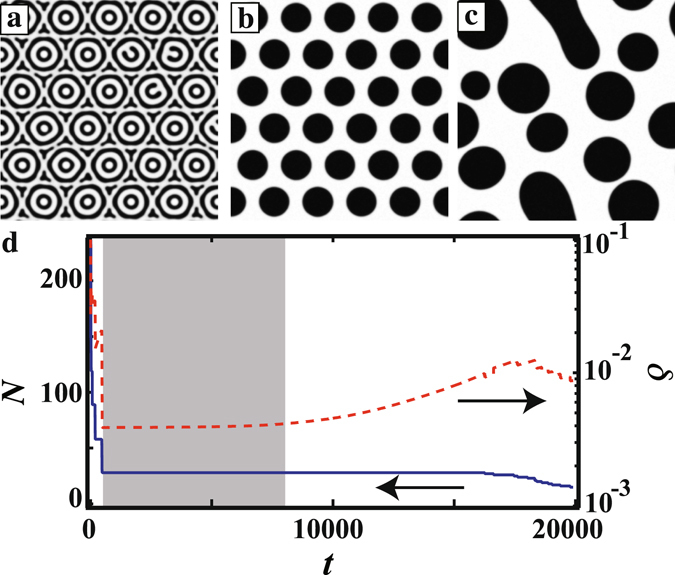
Time evolution of a concentric circle pattern with multiple propagated triggers when Δ*d* = 80, $\overline{\varphi }$ = 0.1, and *v*_*tri*_ = 0.5. (**a**) By *t* = 80, a concentric circle pattern has formed at each core. (**b**) At *t* = 5000, a hexagonal pattern is observed after coarsening of the concentric circles. (**c**) Finally, at *t* = 20000, the hexagonal pattern collapses due to thermal noise. The arrangement and size distribution of the droplets becomes random. (**d**) Time evolution of the number of domains *N* (solid line) and the size distribution of the domain *δ* (dotted line). *N* and *δ* are large at the beginning of the phase separation since concentric circles are formed. *N* and *δ* decrease due to coarsening and a regular hexagonal pattern is formed. *δ* remains constant from *t* = 500 to 8000 (shaded region), showing that the regular hexagonal pattern is stable. After *t* = 8000, the droplet sizes have a narrow distribution due to thermal noise, and *δ* gradually increases. By *t* = 15000, the RHD pattern has collapsed due to coarsening.

We also investigated pattern formation with multiple propagated triggers. The propagating triggers are initiated simultaneously from points arranged in a hexagonal pattern with a spacing Δ*d*. Figure 5(a–c) show the time evolution of the pattern at $\overline{\varphi }$ = 0.1, *v*_*tri*_ = 0.5, and Δ*d* = 80. At the beginning of the phase separation, the CC pattern is formed at every point where the triggers start to propagate, as shown in Fig. 5(a). We find that *ϕ* at the boundary of the neighbouring CC pattern is not changed with time, while *ϕ* at the core oscillates [See Fig. 4(a)]. This oscillation of *ϕ* occurs at the core accompanied by a reduction in the number of rings. The CC pattern goes on to coarsen into a regular hexagonal droplet (RHD) pattern [Fig. 5(b)]. The sizes of the droplets become increasingly widely distributed due to thermal noise after a hexagonal array is produced, finally beginning to coarsen again [Fig. 5(c)]. In addition, in Fig. 5(d), we show the time evolution of the number of domains *N* and the droplet size distribution *δ*. *N* decreases at the beginning of the phase separation due to the coarsening of the circular rings. It then remains constant from *t* = 500 to *t* = 15000 when an RHD pattern has formed. After the RHD pattern begins to collapse, the coarsening proceeds again and the number of droplets decreases. Meanwhile, we also find that *δ* is constant from *t* = 500 to *t* = 8000. This means that the size distribution of the droplets is quite small and thus that the RHD arrangement is stable over time. After *t* = 8000, the droplet size distribution has some width due to thermal noise, and *δ* gradually increases. The size difference induces a coarsening of the RHD patterns. We stress here that the regular hexagonal pattern is metastable for a long time in this simulation.

**Figure 6:**
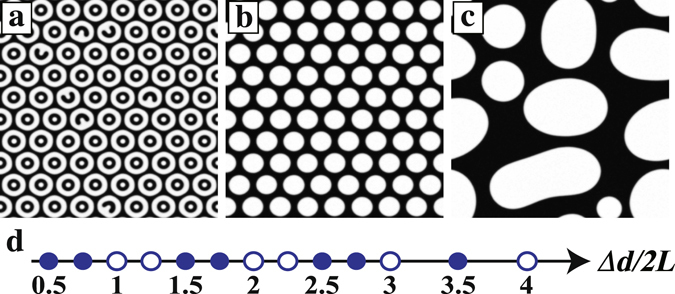
Time evolution of the concentric circle pattern with multiple propagated triggers when Δ*d* = 60, $\overline{\varphi }$ = 0.1, and *v*_*tri*_ = 0.5. (**a**) By *t* = 60, a concentric circle pattern has formed at each core. (**b**) At *t* = 5000, a hexagonal pattern is observed after coarsening of the concentric circles. (**c**) Finally, at *t* = 20000, the hexagonal pattern has collapsed due to thermal noise. The final pattern is the inverse of the pattern when Δ*d* = 60. (**d**) Diagram of the final pattern for different Δ*d*/2*L*. The symbols in (**d**) are the simulated points. A filled circle corresponds to a pattern where droplets of the majority (black) phase form in the minority (white) phase. An open circle corresponds to a pattern where droplets of the minority phase form in the majority phase.

Furthermore, we find that the *ϕ* distribution in the RHD pattern can be controlled by changing Δ*d*. Figure 6(a–c) show the time evolution of the pattern when $\overline{\varphi }$ = 0.1, *v*_*tri*_ = 0.5, and Δ*d* = 60. We observe an inverted pattern compared to what is seen when Δ*d* = 80 [See Fig. 5(a–c)]. The Δ*d* dependence of the RHD patterns can be seen in in Fig. 6(d), where Δ*d*/2*L* corresponds to the wavenumber found from the periodicity of the circular rings, measured from the core to the boundary, where *L* is the pitch length of the circular rings, approximately 20 in this simulation. A filled circle corresponds to a pattern where droplets of the majority (black) phase lie in the minority (white) phase; an open circle corresponds to the opposite, where droplets of the minority phase sit in the majority phase. We find that the pattern oscillates when varying Δ*d*/2*L*. When *n* < Δ*d*/2*L* < *n *+ 0.5 where *n* is an integer, *ϕ* at the boundary of the neighbouring CC pattern is −1. Meanwhile, when *n *+ 0.5 < Δ*d*/2*L* < *n* + 1, *ϕ* at the boundary of the neighbouring CC pattern is 1. Since $\overline{\varphi }$ is conserved, *ϕ* at the core in the final state should be opposite to *ϕ* at the boundary of the neighbouring CC pattern. Thus the *ϕ* distribution of the RHD pattern is determined by *ϕ* at the boundary of the neighbouring CC pattern. We suggest that Δ*d* is a control parameter for the spatial distribution of *ϕ* in the RHD pattern when there are multiple triggers. We note that the majority phase forms droplets when Δ*d* = 60, despite the equilibrium state being one where the minority phase forms droplets instead. Since the energy barrier for reaching the equilibrium state is quite high, the inverted state is stable, even though coarsening occurs. Thus, this simulation also demonstrates how an inverted *ϕ* distribution can be achieved for phase separation.

**Figure 7:**
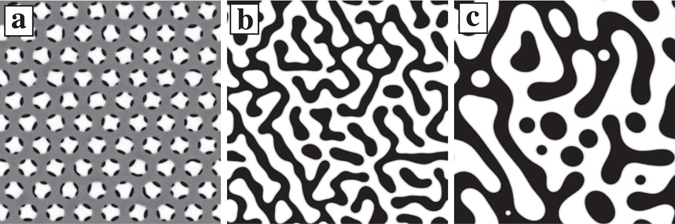
Time evolution of the dendritic pattern with multiple propagated triggers when Δ*d* = 60, $\overline{\varphi }$ = 0.1, and *v*_*tri*_ = 0.01. (**a**) By *t* = 3500, a dendritic pattern has formed at each core. (**b**) At *t* = 50000, a connected pattern is observed. (**c**) The same can be seen when *t* = 100000. Connectivity can be seen for a long time despite the fact that $\overline{\varphi }$ = 0.1.

Finally, we also look at dendritic pattern formation with multiple propagated triggers. Figure 7 show the time evolution of the dendritic pattern when $\overline{\varphi }$ = 0.1, *v*_*tri*_ = 0.01, and Δ*d* = 60 for *t* = (a) 3500, (b) 50000, and (c) 100000. Firstly, a dendritic pattern is formed at each core at *t* = 3500. Next, they come into contact with neighbouring dendritic patterns and the phases connect. By *t* = 50000, the surface has become smooth due to surface energy. We note that the percolated pattern remains for our simulation time, while a random droplet pattern is observed for $\overline{\varphi }$ = 0.1 with homogeneous quenching, similar to that in Fig. 1(a).

## Discussion

Here, we discuss the difference between pattern formation observed in our trigger propagation system and in surface directed spinodal decomposition. Firstly, surface directed spinodal decomposition requires one component to be preferentially attracted to a wall, while pattern formation by trigger propagation can be observed even though both components have the same wetting properties to the wall. In addition, the time evolution of the concentric circles is also different. The concentric circles coarsen without changing the phase at the core in surface directed spinodal decomposition; meanwhile, with multiple triggers, the phase at the core of the circles changes repeatedly, allowing us to control which phase takes on the form of droplets [See Figs 5 and 6].

We also note that a regular droplet pattern may have noteworthy physical properties such as optical response, electrical response etc. Furthermore, despite bicontinuous structures being associated with nearly symmetric compositions, we have shown that this can be achieved even with asymmetric composition, with multiple triggers propagating through the system. A bicontinuous pattern entails percolation of both components, leading to its own set of unique physical properties. We can thus hope that our simulation results motivate more experiments and understanding of the physics of pattern structure and formation.

## Summary

To summarize, we performed numerical simulations with propagated triggers which induce phase separation. The trigger induces the effects of a temperature quench in real systems. We investigate pattern formation for different trigger propagation speeds and mean area fraction. We find a random droplet pattern, a concentric circle pattern, and a dendritic pattern. In the concentric circle pattern, *ϕ* at the core oscillates during the coarsening process, while *ϕ* at the boundary of a neighbouring concentric circle pattern is unchanged. We then investigated pattern formation with multiple propagated triggers. A long-ranged hexagonal droplet pattern is formed when we propagate the triggers from hexagonally arranged points. In addition, we find that the *ϕ* distribution over space can be controlled by changing the spacing of the hexagonal arrangement. This simulation not only demonstrates a way in which long-ranged regular patterns can be formed, but also how one might invert the spatial *ϕ* distribution of the phase separated system. We also demonstrate formation of a percolated pattern when $\overline{\varphi }$ = 0.1 with slower propagated triggers. Although our model is minimal in construction, similar results may occur in realistic systems through much more complex means. We conclude that pattern formation can be significantly influenced by changing the arrangement of the triggers, and believe that our study demonstrates an effective means by which pattern formation can be controlled in phase separating systems.

## References

[1] Cahn JW, Hilliard JE (1958). Free energy of a nonuniform system. i. interfacial free energy. J. Chem. Phys..

[2] Oono Y, Puri S (1987). Computationally efficient modeling of ordering of quenched phases. Phys. Rev. Lett..

[3] Puri S (1988). Phase separation in an off-critical quench. Phys. Lett. A.

[4] Onuki, A. *Phase Transition dynamics* (Cambridge University Press, 2002).

[5] Tanaka H, Araki T (2000). Simulation method of colloidal suspensions with hydrodynamic interactions: Fluid particle dynamics. Phys. Rev. Lett..

[6] Tanaka H, Araki T, Koyama T, Nishikawa Y (2005). Universality of viscoelastic phase separation in soft matter. J. Phys. Condens. Matter.

[7] Hamley, I. W. *Introduction to soft matter: synthetic and biological self-assembling materials* (John Wiley & Sons., 2007).

[8] Connor MT, Roy S, Ezquerra TA, Calleja FJB (1998). Broadband ac conductivity of conductor-polymer composites. Phys. Rev. B.

[9] Ball RC, Essery RLH (1990). Surface-directed spinodal decomposition: modelling and numerical simulations. J. Phys.: Condens. Matter.

[10] Berthier L (2001). Phase separation in a homogeneous shear flow: Morphology, growth laws, and dynamic scaling. Phys. Rev. E.

[11] Golovin AA, Nepomnyashchy AA, Davis SH, Zaks MA (2001). Convective cahn-hilliard models: From coarsening to roughening. Phys. Rev. Lett..

[12] Tanaka H (2000). Viscoelastic phase separation. J. Phys. Condens. Matter.

[13] Puri S, Binder K (1992). Surface-directed spinodal decomposition: phenomenology and numerical results. Phys. Rev. A.

[14] Puri S, Frisch HL (1997). Surface-directed spinodal decomposition: modelling and numerical simulations. Journal of Physics: Condensed Matter.

[15] Kielhorn L, Muthukumar M (1999). Phase separation of polymer blend films near patterned surfaces. J. Chem. Phys..

[16] Yan L-T, Li J, Li Y, Xie X-M (2008). Kinetic pathway of pattern-directed phase separation in binary polymer mixture filmes. Macromolecules.

[17] Krekhov A, Weith V, Zimmermann W (2013). Periodic structures in binary mixtures enforced by janus particles. Phys. Rev. E.

[18] Iwashita Y, Kimura Y (2013). Stable cluster phase of janus particles in two dimensions. Soft Matter.

[19] Tanaka H, Sigehuzi T (1995). Periodic spinodal decomposition in a binary polymeric fluid mixture. Phys. Rev. Lett..

[20] Henderson IC, Clarke N (2004). Two-step phase separation in polymer blends. Macromolecules.

[21] Singh A, Mukherjee A, Vermeulen HM, Barkema GT, Puri S (2011). Control of structure formation in phase-separating systems. J. Chem. Phys..

[22] Jaiswal PK, Puri S, Binder K (2013). Phase separation in thin films: Effect of temperature gradients. Eur. Phys. Lett..

[23] Furukawa H (1992). Phase separation by directional quenching and morphological transition. Physica A.

[24] Furukawa H (1994). Concentric patterns in mesoscopic spinodal decomposition. J. Phys. Soc. J..

[25] Liu B, Zhang H, Yang Y (2000). Surface enrichment effect on the morphological transitions induced by directional quenching for binary mixtures. J. Chem. Phys..

[26] Krekhov A (2009). Formation of regular structures in the process of phase separation. Phys. Rev. E.

[27] Hashimoto T, Bodycomb J, Funaki Y, Kimishima K (1999). The effect of temperature gradient on the microdomain orientation of diblock copolymers undergoing an order-disorder transition. Macromolecules.

[28] Okada M, Masunaga H, Furukawa H (2000). Concentric pattern formation during phase separation induced by a cross-linking reaction. Macromolecules.

[29] Cook HE (1970). Brownian motion in spinodal decomposition. Acta Metall..

[30] Oikawa N, Kurita R (2016). A new mechanism for dendritic pattern formation in dense systems. Sci. Rep..

